# A Review of Wireless Sensor Technologies and Applications in Agriculture and Food Industry: State of the Art and Current Trends

**DOI:** 10.3390/s90604728

**Published:** 2009-06-16

**Authors:** Luis Ruiz-Garcia, Loredana Lunadei, Pilar Barreiro, Jose Ignacio Robla

**Affiliations:** 1 Laboratorio de Propiedades Físicas y Tecnologías Avanzadas en Agroalimentación, Universidad Politécnica de Madrid, / ETSI Agrónomos, Edificio Motores, Avda. Complutense s/n 28040 Madrid, Spain; E-Mails: loredana.lunadei@gmail.com (L.L.); pilar.barreiro@upm.es (P.B.); 2 Centro Nacional de Investigaciones Metalúrgicas (CENIM-CSIC), / Avda. Gregorio del Amo, 8, 28040, Madrid, Spain; E-Mail: jrobla@cenim.csic.es

**Keywords:** WSN, RFID, agriculture, food

## Abstract

The aim of the present paper is to review the technical and scientific state of the art of wireless sensor technologies and standards for wireless communications in the Agri-Food sector. These technologies are very promising in several fields such as environmental monitoring, precision agriculture, cold chain control or traceability. The paper focuses on WSN (Wireless Sensor Networks) and RFID (Radio Frequency Identification), presenting the different systems available, recent developments and examples of applications, including ZigBee based WSN and passive, semi-passive and active RFID. Future trends of wireless communications in agriculture and food industry are also discussed.

## 1. Introduction

Wireless Sensor Technologies (WST) are entering a new phase. Recent advances offer vast opportunities for research and development. This is the consequence of the decreasing costs of ownership, the engineering of increasingly smaller sensing devices and the achievements in radio frequency technology and digital circuits.

WST refers to Wireless Sensor Networks (WSN) and radio frequency identification (RFID) based sensor devices. WSN is one of the most significant technologies in the 21st century. RFID was developed for identification purposes, but growing interest in the many other possible applications has led to the development of a new range of wireless sensor devices based on RFID. The main difference between a WSN and a RFID system is that RFID devices have no cooperative capabilities, while WSN allow different network topologies and multihop communication.

These technologies have been attracting many research efforts during the past few years, driven by the increasing maturity and adoption of standards, such as Bluetooth [1] and ZigBee [2] for WSN, and various ISO (International Organization for Standards) standards for RFID (ISO 15693, ISO/IEC 18000, ISO 11784, etc.) [3-9]. Currently, they are very promising in several fields such as environmental monitoring, irrigation, livestock, greenhouse, cold chain control or traceability. The systems are usually composed of a few sinks and large quantity of small sensors nodes. Normally, these sensor nodes consist of three components: sensing, processing and communicating [10]. Each wireless sensor node communicates with a gateway unit which can communicate with other computers via other networks, such as a Local Area Networks (LAN), Wireless Local Area Networks (WLAN) [11,12], Internet [13], Controller Area Network (CAN) [14] or Wireless Wide Area Network (WWAN) using standard protocols like GSM (Global System for Mobile communication) [15-17] or GPRS (General Packet Radio Service) [18,19].

In this paper, we review the standards and the numerous applications that utilize WST in agriculture and food industry and to classify them in appropriate categories. The analysis of their characteristics and contributions could be useful for perceiving new applications or relevant research opportunities.

## 2. Wired *vs.* Wireless

WSN can operate in a wide range of environments and provide advantages in cost, size, power, flexibility and distributed intelligence, compared to wired ones. In a network, when a node cannot directly contact the base station, the message may be forwarded over multiple hops. By auto configuration set up, the network could continue to operate as nodes are moved, introduced or removed. Monitoring applications have been developed in medicine, agriculture, environment, military, machine/building, toys, motion tracking and many other fields. Architectures for sensor networks have been changing greatly over the last 50 years, from the analogue 4-20 mA designs to the bus and network topology of today. Bus architectures reduce wiring and required communication bandwidth. Wireless sensors further decrease wiring needs, providing new opportunities for distributed-intelligence architectures [2,10,20].

For fieldbus architecture, the risk of cutting the bus that connects all the sensors persists. WSN eliminates all the problems arising from wires in the system. This is the most important advantage of using such technology for monitoring.

Wireless sensor technology allows Micro-Electro-Mechanical Systems Sensors (MEMS) to be integrated with signal conditioning and radio units to form “motes” – all for a low cost, a small size, and with low power requirements. Available MEMS include inertial, pressure, temperature, humidity, strain-gage, and various piezo and capacitive transducers for proximity, position, velocity, acceleration and vibration measurements [20]; and according to several research works, connecting wires to these devices can be more problematic than doing it by means of wireless designs [21,22].

Motes can form networks and co-operate according to various models and architectures. They came with miniaturized sensors mounted, that allow, in a small space (2.5 × 5 × 5cm), the gathering of data not only just about temperature, but also relative humidity, acceleration, shock and light [23].

Another advantage for wireless sensor devices is the feasibility of installation in places where cabling is impossible, such as large concrete structures [24] or embedded within the cargo, which brings their readings closer to the true in situ properties of perishable products [25].

Wired networks are very reliable and stable communication systems for instruments and controls. However, wireless technology promises lower installation costs than wired devices, because required cabling engineering is very costly [26].

## 3. Wireless Sensor Networks

A WSN is a system comprised of radio frequency (RF) transceivers, sensors, microcontrollers and power sources [10]. Recent advances in wireless sensor networking technology have led to the development of low cost, low power, multifunctional sensor nodes. Sensor nodes enable environment sensing together with data processing. Instrumented with a variety of sensors, such as temperature, humidity and volatile compound detection, allow monitoring of different environments. They are able to network with other sensor systems and exchange data with external users [27].

Sensor networks are used for a variety of applications, including wireless data acquisition, machine monitoring and maintenance, smart buildings and highways, environmental monitoring, site security, automated on-site tracking of expensive materials, safety management, and in many other areas [10].

A general WSN protocol consists of the application layer, transport layer, network layer, data link layer, physical layer, power management plane, mobility management plane and the task management plane [10].

Currently two there standard technologies are available for WSN: ZigBee and Bluetooth. Both operate within the Industrial Scientific and Medical (ISM) band of 2.4 GHz, which provides license-free operations, huge spectrum allocation and worldwide compatibility. In general, as frequency increases, bandwidth increases allowing for higher data rates but power requirements are also higher and transmission distance is considerably shorter [27,28]. Multi-hop communication over the ISM band might well be possible in WSN since it consumes less power than traditional single hop communication [28].

It is also possible to create a WSN using Wi-Fi (IEEE 802.11), but this protocol is usually utilized in PC-based systems because it was developed to extend or substitute for a wired LAN [29]. Its power consumption is rather high, and the short autonomy of a battery power supply still remains an important disadvantage [30].

### Bluetooth

Bluetooth (IEEE 802.15.1) was developed as a wireless protocol for short-range communication in wireless personal area networks (PAN) as a cable replacement for mobile devices. It uses the 868 and 915 MHz and the 2.4 GHz radio bands to communicate at 1 Mb per second between up to seven devices (see Table 1). Bluetooth is mainly designed to maximize ad hoc networking functionality. Some of its common functions are passing and synchronizing data, e.g. between a PDA (personal digital assistant) and a computer, wireless access to LANs, and connection to the internet. It uses frequency-hopping spread-spectrum (FHSS) communication, which transmits data over different frequencies at different time intervals. Bluetooth uses a master-slave-based MAC (medium access control) protocol [1,31,32].

### ZigBee

The ZigBee standard is built on top of the IEEE 802.15.4 standard. The IEEE 802.15.4 standard defines the physical and MAC (Medium Access Control) layers for low-rate wireless personal area networks [33]. The physical layer supports three frequency bands with different gross data rates: 2,450 MHz (250 kbs^-1^), 915 MHz (40 kbs^-1^) and 868 MHz (20 kbs^-1^). It also supports functionalities for channel selection, link quality estimation, energy measurement and clear channel assessment. ZigBee standardizes both the network and the application layer. The network layer is in charge of organizing and providing routing over a multi-hop network, specifying different network topologies: star, tree, peer-to-peer and mesh. The application layer provides a framework for distributed application development and communication.

Aside from the agriculture and food industry, it is widely used in home building control, automation, security, consumer electronics, personal computer peripherals, medical monitoring and toys. These applications require a technology that offers long battery life, reliability, automatic or semiautomatic installation, the ability to easily add or remove network nodes, signals that can pass through walls and ceilings and a low system cost [2].

### Bluetooth vs. ZigBee

Table 1 provides a comparison between ZigBee and Bluetooth. For applications where higher data rates are important, Bluetooth clearly has the advantage since it can support a wider range of traffic types than ZigBee. However, the power consumption in a sensor network is of primary importance and it should be extremely low [28]. Bluetooth is probably the closest peer to WSNs, but its power consumption has been of secondary importance in its design. Bluetooth is therefore not suitable for applications that require ultra-low power consumption; turning on and off consumes a great deal of energy. In contrast, the ZigBee protocol places primary importance on power management; it was developed for low power consumption and years of battery life. Bluetooth devices have lower battery life compared to ZigBee, as a result of the processing and protocol management overhead which is required for ad hoc networking [28,34]. Also, ZigBee provides higher network flexibility than Bluetooth, allowing different topologies. ZigBee allows a larger number of nodes – more than 65,000 – according to specification. Thus, the suitability of ZigBee for monitoring in agriculture and food industry has been proposed by various authors [14,28,34-36].

## 4. Radio Frequency Identification

RFID is an emerging technology that makes use of wireless communication. The protocol was originally developed for short-range product identification, typically covering the 2 mm - 2 m read range, and has been promoted as the replacement technology for the optical bar-code found, with the use of EPC (Electronic Product Code). RFID has the ability to allow energy to penetrate certain goods and to read a tag that is not visible [37].

There are many distinct protocols used in the various RFID systems, some using the lower end of the spectrum (135 KHz) and others using the super high frequency (SHF) at 5.875 GHz:

There are various standards involved in RFID:

- ISO/IEC 7816 is the standard for contact chip cards [6].
- ISO/IEC 14443 is for contactless proximity cards operating at 13.56 MHz [7].
- ISO/IEC 15693 is for contactless vicinity cards operating at 13.56 MHz [8].
- ISO/IEC 18000 is for item management air interface, defining the parameters for air interface in different frequencies: < 135 kHz, 13.56 MHz, 2.45 GHz, 5.8 GHz, 860-930 MHz and 433 MHz [9].
- ISO 11784, ISO 11785 and ISO 14223 are standards for the radio-frequency identification of animals [3-5].

RFID systems are comprised of three main components: the tag or transponder, the reader or transceiver that reads and writes data to a transponder, and the computer containing database and information management software [38]. RFID tags can be active, passive or semi-passive. Passive and semi-passive RFID send their data by reflection or modulation of the electromagnetic field that was emitted by the Reader. The typical reading range is between 10 cm and 3 m. The battery of semi-passive RFID is only used to power the sensor and recording logic. The communication of active RFID is powered by his own battery. This enables higher signal strength and extended communication range of up to 100 meters; but the implementation of active communication requires larger batteries and more electronic components. The typical price of active RFID is between five or ten times the price of semi-passive ones [39].

RFID has been successfully applied to food logistics and supply chain management processes because of its ability to identify, categorize, and manage the flow of goods [39-42]. Also, electronic identification of cattle using RFID is a common practice in many farms [43]. However, recent developments in RFID hardware outfitted with sensors extend its range of application.

Murkovic *et al*. developed a RFID passive tag with a chemical sensor and its optoelectronic interface. The device is battery-free, has the size of a credit card and is compatible with the ISO 15693. It measures pH in the range 5.0-8.5, using wireless energy transfer to power the sensor and read its response [44].

There are commercial active and semi-passive tags that can collect temperature information [45,46]. Other semi-passive tags outfitted with sensor are under development, like humidity [47,48], shock/vibration [49], light [48,50] and concentration of gases, such as acetaldehyde or ethylene [51].

In agriculture, active tags are very interesting, especially for animal behavior studies. They automatically send impulses, so the animals can be identified by even distant readers. This ability is guaranteed by using a power battery. These devices can be used to monitor animals in mid-size outdoor pens, providing digital data that can be easily computerized [52].

## 5. WST Applications

This section presents most relevant applications in agriculture and food industry. The development of these applications in agro-food has attracted considerable research efforts in the last years, because these technologies are very suitable for distributed data collecting and monitoring in tough environments such as greenhouses, cropland, warehouses or refrigerated trucks. However, some areas have been developed faster than others. For example, there are several applications for monitoring greenhouses or livestock and just a few in farm machinery.

### 5.1. Physical Aspects of Applying WST in Agriculture and Food Industry

Radio propagation in real environments is complex due to multipath propagation, shadowing and attenuation. In agriculture, the radio frequency faces challenges due to placement of nodes for wide-area mesh coverage and reliable link quality above crop canopies. WST must be able to operate in a wide range of environments such as bare fields, vineyards, orchards, from flat to complex topography and over a range of weather conditions, all of which affect radio performance [53]. In these situations, the link power budget is dependent on crop growth and terrain in addition to more common factors such as node spacing and antenna height [54]. For applications inside buildings like burns, greenhouses or warehouses, the radio signal has to go through many objects like walls, windows, pallets, machines, etc. which also cause a significant reduction in signal strength. In general, a received signal level of 10 dB to 20 dB above the sensitive limit of the receiver is an accepted value for the link budget [54].

One important research topic is fault detection and isolation. In a remote sensing application it is essential to detect the erroneous measurements. Wrong information provided by the monitoring system should be identified and skipped. Also the implementation of artificial intelligence in the core of the system can block the transmission of erroneous data. Ruiz-Garcia *et al*. found that sensor measurements become erroneous when the battery voltage was low, between 2,159-2,167 mV (full charge was 3,000 mV) temperature rises enormously and both relative humidity and temperature increase in variability [25]. However, this behavior has not been documented by other studies in other fields that use the same motes [55,56]. Thus the possible influence of other factors like the algorithm installed should be taking into account and studied in further experiments.

Among the different performance parameters, the rate of message lost or Packet Reception Rate (PRR) is very important in a WST implementation, and should be evaluated for any application. Depending on the operating environment, significant signal loss can occur at these frequencies particularly when the radios require line-of-sight for optimal performance, with 2.4 GHz more susceptible than 900 MHz. Results from experimentation in refrigerated chambers with 2.4 GHz ZigBee motes showed low ratio of data lost packets. Being the maximums detected 15.73% at the laboratory and 4.74% at the wholesale store. In opposition, the ratio founded in a semi-trailer, had a maximum of 100% for two of the motes and a minimum of 32.48% [57]. In the case of Ipema *et al*., who monitored cows with 433 MHz motes, the base station directly received less than 50% of temperature measurements stored in the mote buffer [56]. Nadimi *et al*., that also monitored cows with 2.4 GHz motes, showed packet loss rates of about 25% for wireless sensor data from cows in a pasture when the distance to the receiver was less than 12.5 m. In a potato field, after the first year of experimentation with 868/916 MHz, 98% of data packets were lost. However, during the second year the total amount of data gathered was 51%, which represents a clear improvement [58,59]. Looking for better results, a solution could be to display more intermediates motes that allow peer to peer communication to the base station. Another solution could be to do more experiments testing other frequencies like 868 and 916 MHz, which are used for other commercial motes available. Or to develop motes with more RF power, that can achieve longer radio ranges. Also the transmission could be improved by means of optimizing antenna orientation, shape and configuration [57,60].

#### Climate Influence

Signal loss due to atmospheric conditions should be considered because the climate does influence the communication links [54]. Outdoor use has to take into account the effects of moisture due to humidity, precipitation and wetting. Goense and Thelen studied the propagation of radio waves in a potato field using 433 MHz motes, finding a better propagation under wet conditions. Higher relative humidity and rain provided increased signal strength at the receiver [12]. However, experimentation with 868/916 MHz motes showed the opposite behavior, when the relative humidity during the day was high, the gateway received around 60% of the expected messages. This percentage grows back to more than 70% in the drier days [59]. Other authors, calculated the attenuation of 2.4 GHz signals due to rain as 0.02 dB/km for a rain rate of 150 mm/hr [10].

Also, ambient temperature affects the motes performance. Low temperatures have a negative effect in the battery life of the motes. Experimentation in refrigerated chambers at different temperatures showed that battery life decreases under cooling conditions. For example, in 2.4 GHz ZigBee motes, battery life at 0 °C is half than that at 20 °C [25]. Measurements in motes can become erroneous when the battery voltage is less than a certain threshold. Ruiz Garcia *et al*. reported erroneous data below 2,160 mV compared to 3,000 mV corresponding to full charge. Thus, changes in battery voltage must be isolated from impacting measurement accuracy [25].

#### Crop Canopy Influence

Another factor that changes over time is the density of the leaves in the crop. Signal propagation above the cross canopy results in attenuation and variance in the received signal strength [59,61]. The density of the leaves in the crop changes over time. When there are less leaves the message rate increases. Zhang studied the transmission range of a 2.4 GHz Bluetooth device, finding the optimal distances with radio heights of 1.4 m, 1.7 m and 4 m across bare soil, soybeans and corn respectively [62]. Goense and Thelen found that the weakest signals were measured in July, when the total crop canopy was fully developed. Results showed that a dry, full developed crop canopy limits the distance that radio's can cover to around 11 meters when placed near the soil surface. Thus, 100 motes per hectare would be necessary for a reliable communication over the crop canopy [12]. Hebel *et al*. showed that attenuation and signal strength variance were dependent on line of sight losses and heights less than the Fresnel zone radius [63]. Experimentation in mature corn fields (2.5 m high) with transceivers placed at antenna heights of 1.5 and 2 m. and a distance of 100 m, showed an average 10 dB loss when the transceivers were placed in or across the corn rows [54].

### 5.2. Environmental Monitoring

WSN become an important issue in environmental monitoring. The relatively low cost of the devices allow the installation of a dense population of nodes that can adequately represent the variability present in the environment. They can provide risk assessment information, like for example alerting farmers at the onset of frost damage and providing better microclimate awareness.

#### Climate Monitoring

The automation of the monitoring process can be used in diverse types of climates and conditions. Johnson and Margalho monitored agroclimate in the Amazon, analyzing WSN short range transmission. They found that more distant nodes suffered a performance loss, while nodes closer to the sink maintained their throughput levels. Another example of climate supervision is flood prediction by means of wireless sensors, which can detect rainfall, water level and weather conditions. The sensors supply information to a centralized database system [64]. Pierce and Elliot extended the implementation to a regional and on-farm sensor networks at 900 MHz that provide remote, real-time monitoring and control of farming operations in two agricultural applications, a weather monitoring network and an on-farm frost monitoring network [65].

Ayday and Safak presented a moisture distribution map obtained through the integration of WSN with GIS (Geographic Information Systems). The wireless nodes with moisture sensors were located at predetermined locations; geographic coordinates of these points were obtained with GPS and then, all the information was evaluated using GIS [66].

Han *et al*. developed a wireless data transmission system, using wireless ZigBee motes, developed to remotely monitor sediment runoff at a low-water crossing in real time. The gateway transmitted the sensor signals to an Internet server using the GPRS [18].

Hamrita and Hoffacker developed a lab prototype for wireless measurement of soil temperature. The system was based in a commercial 13.56 MHz RFID tag. Measurements showed a high correlation (greater than 99%) with those obtained using a thermocouple [67].

#### Fire Detection

Current surveillance systems use a camera, an infrared sensor system and a satellite system. These systems cannot support real-time surveillance, monitoring and automatic alarm. A wireless sensor network can detect and forecast forest fire more promptly than the traditional satellite-based detection approach. WSN based fire surveillance systems was designed and implemented. WSN measure temperature and humidity, and detect smoke [68,69].

### 5.3. Precision Agriculture

**Figure 1.**
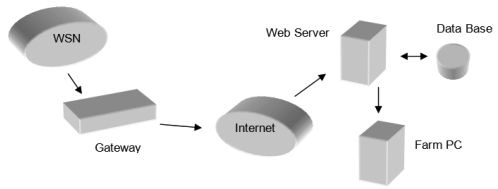
Proposal of remote sensing architecture in precision agriculture.

The development of WST applications in precision agriculture (see Figure 1) makes possible to increase efficiencies, productivity and profitability while minimizing unintended impacts on wildlife and the environment, in many agricultural production systems. The real time information from the fields will provide a solid base for farmers to adjust strategies at any time. Instead of take decisions based in some hypothetical average condition, which may not exist anywhere in the reality, a precision farming approach recognizes differences and adjusts management actions accordingly [70].

#### Farm Machinery

WSN implemented in off-road vehicles, such as tractors or combine harvester, allow exchanging data with static infrastructure or with other vehicles, creating of mobile WSN. However, up to date there are no commercial systems available and just a few research studies reported. Lee *et al*. installed a capacitance type moisture sensor in a forage harvester for monitoring moisture concentration during harvesting. The information was transmitted using Bluetooth [71]. Cugati *et al*. reported a Blueooth network deployed in a fertilizer applicator that allowed real-time sensor acquisition. The information was used for calculating the optimal quantity and spread pattern for a fertilizer [72].

#### Pest Control

Baggio deployed a WSN for fighting *phytophtora* in a potato field. *Phytophtora* is a fungal disease which depends on the climatological conditions. 868/916 MHz motes were used for measuring humidity and temperature. The aim of the system is to reveal when the crop is at risk and let the farmer treat the plants only when is really needed [58].

#### Viticulture

Plant monitoring, also called phytomonitoring, is easier using WST. For example, with the help of WST the owner of vineyard can manage the vineyard works more efficiently and automatically. Burrell *et al*. described a variety of sensor network configurations and applications that can address different priorities in the vineyard [73].

Beckwith *et al*. implemented a WSN in a vineyard consisted of 65 motes of 916 MHz. Temperatures measurements were collected during one month. The information was used for addressing two important parameters in wine production: heat summation and potential frost damage [74].

Morais *et al*. shown the feasibility of a ZigBee based remote sensing network, intended for precision viticulture. The network nodes were powered by batteries that are recharged with energy harvested from the environment [75].

#### Precision Irrigation

Efficient water management is a major concern in many crop systems. WST have a big potential for represent the inherent soil variability present in fields with more accuracy than the current systems available. Thus, the benefit for the producers is a better decision support system that allows to maximize their productivity while saving water. Also, WST eliminates difficulties to wire sensor stations across the field and reduces maintenance cost. Since installation of WST is easier than existing wired solutions, sensors can be more densely deployed to provide local detailed data. Instead than irrigating an entire field in response to broad sensor data, each section could be activated based on local sensors.

O'Shaughnessy and Evett used a six-span center pivot irrigation system as a platform for testing two WSN of infrared thermometers. Comparing the performance of a mesh and non-mesh networking systems of wireless sensors on a center pivot platform [76].

Vellidis *et al*. developed a prototype of smart sensor array for scheduling irrigation in cotton. The system integrates moisture sensors, thermocouples and RFID tags [77].

Qian *et al*. designed a new groundwater-monitoring instrument based on WSN. The new instrument monitors groundwater table and temperature through a sensor. An embedded singlechip processes the monitoring data and a GSM data module transfers the data wirelessly [16]. Bogena *et al*. evaluated a low-cost soil water content sensor focusing in it wireless network application [78].

Kim *et al*. develop an in-field WSN for implementing site specific irrigation control in a linear move irrigation system. Communication signals from the sensor network and irrigation controller to the base station were successfully interfaced using low-cost Bluetooth wireless radio communication [79].

Akyildiz and Stuntebeck reported an underground system for monitoring soil conditions, such as water and mineral content, in order to provide data for appropriate irrigation and fertilization. Also, the system can be used for monitoring the presence and concentration of various toxic substances in soils near rivers and aquifers, where chemical runoff could contaminate drinking water supplies. Another application can be landslide prediction by monitoring soil movement [80].

#### Greenhouses

The automation and efficiency on greenhouse environment monitoring and control are crucial. In order to control and monitor the environmental factors, sensors and actuators are essential. Greenhouse crops can benefit a lot using WST, because inside the greenhouse the crop conditions such as climate and soil do not depend on natural agents. Thus, the implementations are easier than in outdoor applications.

The first application of WSN in a greenhouse was reported in the year 2003, it was a monitoring and control system developed by means of Bluetooth [81]. Since that year, several applications has been developed, most of them makes use of IEEE 802.15.4/ZigBee:

Gonda and Cugnasca presented a proposal of a distributed greenhouse control and monitoring system using ZigBee [82]. Yoo *et al*. describes the results of real deployment of a WSN IEEE 802.15.4 compliant to monitor and control the environment in greenhouses with melon and cabbage [11].

Lea-Cox *et al*. developed a WSN in a greenhouse, that integrates a variety of sensors which can measure substrate water, temperature, electrical conductivity, daily photosynthetic radiation and leaf wetness in real-time. Benefits came from an improved plant growth, more efficient water and fertilizer applications, together with a reduction in disease problems related to over-watering [13].

Liu *et al*. reported a WSN prototype with two-part framework for greenhouses. In the first part, several sensor nodes were used to measure temperature, light and soil moisture. The other part consists of GSM module and the management software based on database running on the remote PC [15].

Zhou *et al*. designed a monitoring system based on ZigBee, using an star network topology inside the greenhouse and a mesh topology for the connection between the greenhouses and the management system [19].

Yang *et al*. reported a multi-functional remote sensing system that integrates RFID technology with spectral imaging and environmental sensing in a greenhouse. The multi-spectral imaging system was used for remote sensing of the canopy of cabbage seedlings. Greenhouse temperature, relative humidity, and lighting conditions were measured above the crop [83].

Wang *et al*. develop an specialized wireless sensor node for monitoring temperature, relative humidity and light inside greenhouses [84].

### 5.4. Precision Livestock

Modern animal production has changed in recent years due to the use of precision tools. Results of recent research have been used as inputs to preventive diagnostics and development of decision-making software in several areas, as well as to predict events.

WST has been used as a new technique for measuring core body temperature that are minimally invasive and provide continuous, remote, real-time information. Together with body temperature WSN can obtain the oxygen saturation of cattle's blood using a pulse oximeter, location (GPS), ambient temperature and respiration [85].

Mayer *et al*. created a wireless sensor network platform for animal health and behavior monitoring. A steer was instrumented with both internal and external sensors, using matchbox sized motes placed inside standard drug release capsules. The nodes monitored the intra-rumenal activity of the steer and communicate wirelessly with each other [60].

Marsh *et al*. implanted an injectable RFID and temperature sensor, into the neck of horses in order to measure body temperature with a unique identity code [86]. Ipema *et al*. described the results of an experiment with a temperature sensor built into a bolus placed in the rumen of a cow. The main objective was to demonstrate that capsule-based wireless technology could work in cattle. The mote in the rumen transmitted data to the mote attached to the front leg of the cow; from there the signal was transmitted to the base station [56].

Evaluation of animal welfare can also be determined by wireless monitoring and enable the producer to make the right decision based on real-time management. Nadimi *et al*. addressed and solved the problem of on-line monitoring of cows in an extended area, using ZigBee based wireless sensor networks. A study of wireless sensor networks applied to the monitoring of animal behaviour in the field is described. The problem of online monitoring of cows' presence and pasture time in an extended area covered by a strip of new grass using wireless sensor networks has been addressed [55,87].

Monitoring and control of the quality of indoor environment is very important for animal health and welfare and directly impacts productivity and quality. Ventilation in the stables must be managed in order to avoid long-term over-critical exposure of the animals to ammonia, causing stress, pour health and reduced productivity. Cai *et al*. presented a wireless, remote query ammonia sensor that can track both low and high concentrations of ammonia [88].

At the same time, ventilation and heating must be minimized in order to save energy while keeping temperatures at an adequate level. Cugnasca *et al*. evaluated the capability and usefulness of WSN applied to monitor environmental variables in an animal housing facility. The nodes were moved through the facility to determine different profiles of temperature, humidity and luminosity [89].

Darr and Zhao develop a wireless data acquisition system for monitoring temperature variations in swine barns [90]. Powered from a single 3.6 V, 1,200 mAhr battery, and configured with a sample rate of 5 minutes the motes had a battery life of 3.5 years. Thus ZigBee motes, were found to be suitable for monitoring in confined animal feeding operations environments [90].

### 5.5. Food Industry

The food industry is nowadays facing critical changes in response to consumer needs, which in addition to health and safety concerns, demand an ever larger diversity of food products with high quality standards.

The quality of these products might change rapidly, because they are submitted to a variety of risks during production, transport and storage that are responsible for material quality losses. Parties involved need better quality assurance methods to satisfy customer demands and to create a competitive point of difference. Successful supply chain logistics calls for automated and efficient monitoring and control of all operations. The monitoring should allow establishing a better knowledge, detecting weakness, and optimizing the whole process, all things that potentially would have a significant impact on the supply chain [91].

Also, there is an increasing demand of traceability in the food chain, statutory requirements are growing stricter and there is increasing pressure to develop standardized traceability systems. From the raw material to the sale of goods, more and more information needs to be gathered and made available. In the next years, the lowering cost of WST will provide the opportunity to track and trace not only large and expensive products, but small and cheap ones, creating a new generation of intelligence products [92]. Moreover, the information gathered by the WST can be linked with a traceability system in each step of the life of the product, “*from farm to fork*” [93].

Products can be tracked and traced from the field to the industry. Anastasi *et al*. designed and implemented a WSN-based system for monitoring the productive cycle of high-quality wine in a Sicilian winery. Nodes were deployed both in the field and in the cellar, where wine aging is performed [30].

WSN was also studied for the supervision of temperature during assessment of canned food sterilization, developing a mathematical model analyzing wireless sensor nodes during the process [94].

#### Cold Chain Monitoring and Traceability

Perishable food products such as vegetables, fruit, meat or fish require refrigerated transports. Therefore, temperature is the most important factor when prolonging the practical shelf life of perishable food products. Studying and analyzing temperature gradients inside refrigeration rooms, containers, and trucks is a primary concern of the industry. The supply chain management of fresh foods requires fast decisions because goods are forwarded within hours after arrival at the distribution center. Appropriate planning calls for more information than that which could be provided by standard RFID tracking and tracing. Quality problems should be detected as quickly as possible, and alarms should be triggered when temperature gradients cross a threshold. Even if direct access to the means of transport is not possible, online notifications offer new opportunities for improved transport planning.

**Figure 2.**
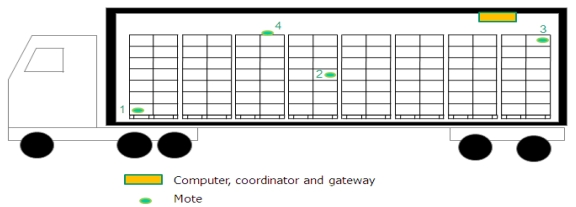
Proposal of WSN inside refrigerated trucks [57].

The use of wireless sensors in refrigerated vehicles was proposed by Qingshan *et al*. [28]. The vehicles can host a variety of sensors to detect, identify, log, and communicate what happens during the journey, monitoring the status of perishable products in transport (see Figure 2). Ruiz-Garcia *et al*. studied and analyzed intermodal refrigerated fruit transport that integrated wireless sensor networks with multiplexed communications, fleet management systems, and mobile networks [14]. ZigBee motes were validated for their use under cooling conditions in warehouses, studying the behavior of the motes in fruit chambers. Also, the psychrometric data model was implemented for quick assessment of changes in the absolute water content of air. Thus it was possible to address water loss from the products, and also to detect condensation on the commodities [25].

The fresh fish logistic chain can be also monitored using WST. Hayes *et al*. reported a WSN based system that allows. The application is built around a web server and bespoke wireless data loggers operating over a GSM network [17]. Abad *et al*. validated a RFID smart tag instrumented with light, temperature and humidity sensors. The system provide real-time traceability information of the product to the different fish distribution chain links [48].

McMeekin used active sensors to record spatial temperature profiles [95]. Gras used passive RFID loggers to test the probability to find a certain temperature in a transport, but did not go into spatial deviations [96]. Amador *et al*. showed the use of RFID for temperature tracking in a commercial shipment of pineapples from Costa Rica to the USA. Jedermann *et al*. monitored 16 refrigerated trucks using semi-passive RFID instrumented with temperature sensors (Turbo Tag) detecting temperature gradients [45]. These kinds of applications can register temperatures during transportation and distribution but the transmission range is less than one meter and they are not able to develop advanced network topologies like the ZigBee devices can do [46]. The accuracy of data loggers is a critical issue in cold chain management. This accuracy becomes even more important if the objective is early detection of temperature changes and gradients. Standards for food distribution allow deviations of ± 0.5°C from the set point [97]. Jedermann *et al*. did a comparison of three different types RFID loggers in a climatic chamber, finding that the percentage of measurements with a difference to the average value less than the deviation ± δ was between 66% and 73% [45].

RFID data loggers are available in high quantities, but they require manual handling because of their low reading range. Another disadvantage is that temperature loggers are only available for the 13.56 MHz HF-Range. The major drawback of this band is the limited reading range of about 20 cm. If a gate reader scans items automatically upon arrival at the warehouse, the reading range has to cover several meters. Also these tags take around five seconds to transfer recorded temperature values over the RFID interface [45]. A high data rate is required according to a normal flow of goods in a warehouse.

Environmental temperature can differs from each other depending in the location of the logger, packing material, or heat dissipation of the product [98,99]. Semi-passive loggers can be also used to measure, not just the walls of the vehicle, but also inside the boxes [46]. There are available commercial solutions for monitoring containers and trucks, but they do not bring complete information about the cargo, because they typically measure in a single or very limited number of points [14].

Craddock and Stansfield proposed sensor fusion for the development of smart containers in order to improve security, gathering data from several sources in order to trigger the alarms. Containers may incorporate a variety of sensors to detect, identify, log and communicate what happens during their journeys around the world [100]. Jedermann *et al*. presented a system for intelligent containers combining wireless sensor networks and RFID [36]. Such devices can be placed in transport vehicles in order to monitor the on-the-go environment and can be the basis for distributed systems, enabling environment sensing together with data processing [14].

In the intermodal transportation, the performance of radio waves inside metal enclosed areas was studied. Furh and Lau tested a RF device in a metal cargo container and demonstrated that it is possible to communicate with the outside world [101]. Laniel *et al*. focuses on the 3-D mapping of RFID signal strength inside a 12 m (40′) refrigerated marine container. Three different types of radio frequency configurations were tested: 2.4 GHz, 915 MHz and 433 MHz. The main goal was to find a frequency and configuration that would allow real time reading of the temperature in a shipment of perishable products using RFID. The results showed a significantly higher performance at the 433MHz level [102].

Appropriated monitoring requires an increasing number of measurements to be performed in food logistics. Specialized WST monitoring devices promise to revolutionize the shipping and handling of a wide range of perishable products giving suppliers and distributors continuous and accurate readings throughout the distribution process. Precise, frequent and automated readings, interpreted by software and coordinated with existing and planned product inventories, should translate into more intelligent goods management and fewer rejected shipments. They could be used to remedy the cause of the problem. But even if a direct access to the means of transport is not possible, online notifications offer new opportunities for improve transport planning. If fixed delivery commitments require ordering of a replacement, the time of information is very crucial.

## 6. Integration of RFID and WSN

After reviewing the applications presented in this paper, one main conclusion is that WSN and RFID are interesting and complementary, because they were originally designed with rather different objectives (RFID for identification while WSN for sensing).

An integration of WSN and RFID allow synergies, WSN uses a variety of sensors like the ones that were mentioned previously, but they cannot identify objects individually while RFID allow the identification of items like container, pallet, boxes or bottles. For this reason, integration of WSN and RFID provides a significant improvement on monitoring and has been faced by recent research.

A possible solution for the integration of RFID, WSN and software agents, in intermodal containers was proposed [36]. The system is called “intelligent container”.

Currently, RFID loggers are roughly 10 times cheaper than wireless sensor nodes (10 € versus 100 €). Both prices will drop; the price of sensor nodes will decrease a bit quicker when their mass production starts, but will always be a multiple of the costs of RFID loggers.

Zhang and Wang describe a deep analysis of RFID and WSN, three forms of new system architecture that combines the two technologies. The first one, mix RFID tags and sensor nodes in the same environment. A station gather information from tags and sensor nodes then transmit it to local host computer or remote server. The second architecture was a new smart node, which makes use of kinds of sensors, to detect interested physical scenario, reading RFID tags, and radio transceiver which transporting sensed data. The last one proposed was to replace the RFID active and semi-active tags by ZigBee motes. The active tag is similar to a mote, but they are not exactly sensor network nodes because they communicate in centralized mode and cannot cooperate with each other through formed ad-hoc networks. Nevertheless, replace all active RFID tags to sensor nodes can be expensive in a large amount of applications [103].

Pereira *et al*. exposed two system architectures to tracking and monitoring animals, integrating WSN and RFID. In both sceneries special wireless sensor nodes that can also read RFID tags are used. This node reads active RFID tags in the long range architecture, while the sorther one passive RFID tag [104].

Sanchez-Lopez and Daeyoung designed an integration scenario where mobile objects and users carrying RFID tags and WSN receive ubiquitous services according to their identity and real-time sensor/actuator information. The main contribution of the proposal was a managed merge of RFID and sensor/actuator information at the context level, as well as a distributed infrastructure that allows the tracking of the context in a real-time manner [105].

## 7. Conclusions

As it was shown, the applications of WSN and RFID are many and varied. The use of WST in agriculture and food industry provides new features that have the potential to be an economically viable replacement to wired networks. The value of technology can be best realized when integrated with agronomic knowledge, using the information gathered in the improvement of decision support systems. Also improving operations by providing early warning of equipment failure and a predictive maintenance tool, improving energy management, providing automatic record-keeping for regulatory compliance, eliminating personnel training costs or reducing insurance costs. The collaboration and synergy of sensing, processing, communication and actuation is the next step to exploit the potential of these technologies.

From 2004 to 2008 the evolution of RFID technology has been developed very fast, adding new features to traditional automatic identification and data capture applications. However, a significant proportion of RFID deployments remain exploratory. Semi-passive tags can be used to monitor environmental variables, such as the temperature, to identify problem areas and to raise alarms. RFID loggers are good tools that are available in high quantities and are cost-effective. However, they require manual handling because of their low reading range.

The main advantages of WSN for monitoring are its longer reading range than RFID, the flexibility and different network topologies that can be configured, the variety of sensors that are already implemented and their low power consumption. Battery life, reliability of measurements and performance in real environments are critical issues that must be improved.

One problem might be that these monitoring systems create huge volumes of data that are difficult to manage, causing a huge increase in the daily volume of data in a corporate information technology system. This increase impacts the hardware cost required for implementing monitoring systems. Neither manual evaluation nor transmission over mobile networks is feasible due to limited bandwidth and expensive usage rates. The solution lies in implementing a decentralized data management system. Data must be pre-processed close to their point of origin by intelligent systems, which could be sited at the level of RFID, motes or the sink.

There is a need to know the long-term behaviour of the systems. Most of the applications reported in this paper have short experimental periods (days or weeks). Longer testing and experimentation is necessary for validate some of applications presented.

Another important benefit of the systems is the visibility that it can give along the food chain. Measurements obtained are consistent and provide valuable information on the conditions encountered during the life cycle of the products. It is possible to address, at regular time increments, what is happening with the product, whether it is temperature, humidity, acceleration, etc. Another advantage is providing effective support in legal situations as well as safety inspections.

The integration of WSN and RFID seems to be a good idea, taking advantage of the complementary features of both technologies. For this purpose, some theoretical approaches have been presented, but a lot of work remains to be done.

## Figures and Tables

**Table 1.** Comparison between Bluetooth and ZigBee.

	**Bluetooth**	**ZigBee**
Standards	IEEE 802.15.1	IEEE 802.15.4
Data rate	1 Mb s^-1^	20-250 kb s^-1^
Latency (time to establish a new link)	< 10 s	30 ms
Frequencies	2.4 GHz	2.4 GHz
No. of nodes	8	65,000
Range	8 m (Class II, III) to 100 m (Class I)	1-100 m
Modulation	FHSS ^2^	DSSS ^1^
Network topology	*Ad hoc* piconets	*Ad hoc*, star, mesh
Data type	Audio, graphics, pictures, files	Small data packet
Battery life	1 week	> 1 year
Extendibility	No	Yes

1 DSSS: Direct Sequence Spread Spectrum.

2 FHSS: Frequency Hopped Spread Spectrum.
